# Factors related to pediatric unintentional burns: the comparison of logistic regression and data mining algorithms

**Published:** 2019-08

**Authors:** Abbas Aghaei, Hamid Soori, Yadollah Mehrabi, Azra Ramezankhani

**Affiliations:** ^*a*^PhD in Epidemiology, Social Determinants of Health Research Center, Research Institute for Health Development, Kurdistan University of Medical Sciences, Sanandaj, Iran.; ^*b*^PhD in Epidemiology, Safety Promotion and Injury Prevention Research Center, School of Public Health, Shahid Beheshti University of Medical, Tehran, Iran.; ^*c*^PhD in Biostatistics, Department of Epidemiology, School of Public Health and Safety, Shahid Beheshti University of Medical Sciences, Tehran, Iran.; ^*d*^PhD in Clinical Science, Prevention of Metabolic Disorders Research Center, Research Institute for Endocrine Science, Shahid Beheshti University of Medical Sciences, Tehran, Iran.

## Abstract

**Background:**

Burn injuries are one of the traumas seen in all parts of the world and children are usually one of the vulnerable groups. The aim of this study was to determine the factors related to unintentional burns in children, using data mining algorithms.

**Methods:**

In this hospital-based case-control study conducted in Kermanshah province, Iran, data were collected over a period of 15 months. Children under the age of 15 years old who were referred to the burn ward of Imam Khomeini Hospital, the only burn referral in Kermanshah province, were included as cases. For the control group, children who were admitted to Dr. Mohammad Kermanshahi Hospital, the only specialist and subspecialist pediatric center in this province, were included. Frequency matching was performed for age and sex. Support vector machine (SVM), artificial neural network (ANN), random forest (RF), and logistic regression (LR) were employed to determine the factors related to burns in children.

**Results:**

The mean age of children with burn injuries was 4.29 ± 3.51 years and 58% of them were boys. The ANN algorithm had better performance than other algorithms. Body mass index (BMI), socioeconomic status, hours without a watchful, mother’s age, mother’s education, household size, father’s job, father’s age, having more than one watchful, and petroleum storage were the most important factors related to pediatric burns.

**Conclusions:**

The majority of the burn-related variables were related to individuals’ social welfare status and their environments. Lessening the effects of these factors could reduce the incidence of pediatric burns.

**Keywords:**

Unintentional burns, Pediatric, Data mining

